# Treatment of impacted ear wax: a case for increased community-based microsuction

**DOI:** 10.3399/bjgpopen20X101064

**Published:** 2020-04-01

**Authors:** Jonathan C Radford

**Affiliations:** 1 GP, Trinity Court Surgery, Stratford-Upon-Avon, UK

**Keywords:** ENT, community care, microsuction, general practice, primary healthcare

## Introduction

Excess earwax is a very common problem, with figures estimating a prevalence of between 700 000 and 2 million adults in England and Wales. Although wax build up can occur in any individual, risk factors include anatomical deformity, hairs in the external canal, physical barriers to wax excretion, dermatological conditions affecting the ear, use of cotton buds, and increasing age (Box 1).^1^ Requests for the removal of ear wax are a very common reason for presentation in primary care. In the US, approximately 150 000 wax removals are performed each week.^2^ A survey of GPs in Edinburgh by Sharp *et al* put estimates at nine patients per month per doctor, equating to two patients per week.^3^ A more recent study suggested that 2.3 million ear irrigations are performed in England and Wales every year.^4^

Box 1 **Risk factors for cerumen impaction**

**Table:** 

**Risk factors for cerumen impaction**
**Anatomical**	Narrow/stenotic external ear canalOsteoma/exotoses of external ear canalHair in external ear canal
**Physical barriers**	Hearing aidsEar plugsCotton bud use (pushes wax further into canal)
**Wax properties**	Hard wax producersDry wax (age-related: cerumen glands atrophy)Otitis externa
**Learning disability**	
**Dermatological conditions**	Eczema, psoriasis, seborrhoeic dermatitis

Funding for treatment of impacted earwax is variable across clinical commissioning groups (CCGs). National Institute for Health and Care Excellence(NICE) guidance recommends ear wax removal should be performed in primary care,^5^ although without a commissioned service, GPs are under no obligation to do so. The funding for these additional services is variable by CCG area, with many practices now opting out of providing irrigation due to cost, high service demand, and safety implications. High set up cost and training requirements make provision of microsuction at individual practice level unfeasible, however many CCGs have funded community-based microsuction services. Access is usually restricted based on NICE criteria for onward referral, and referral to local hospital-based microsuction may also be restricted in parallel to this.

### Treatment approaches

**Figure 1.**
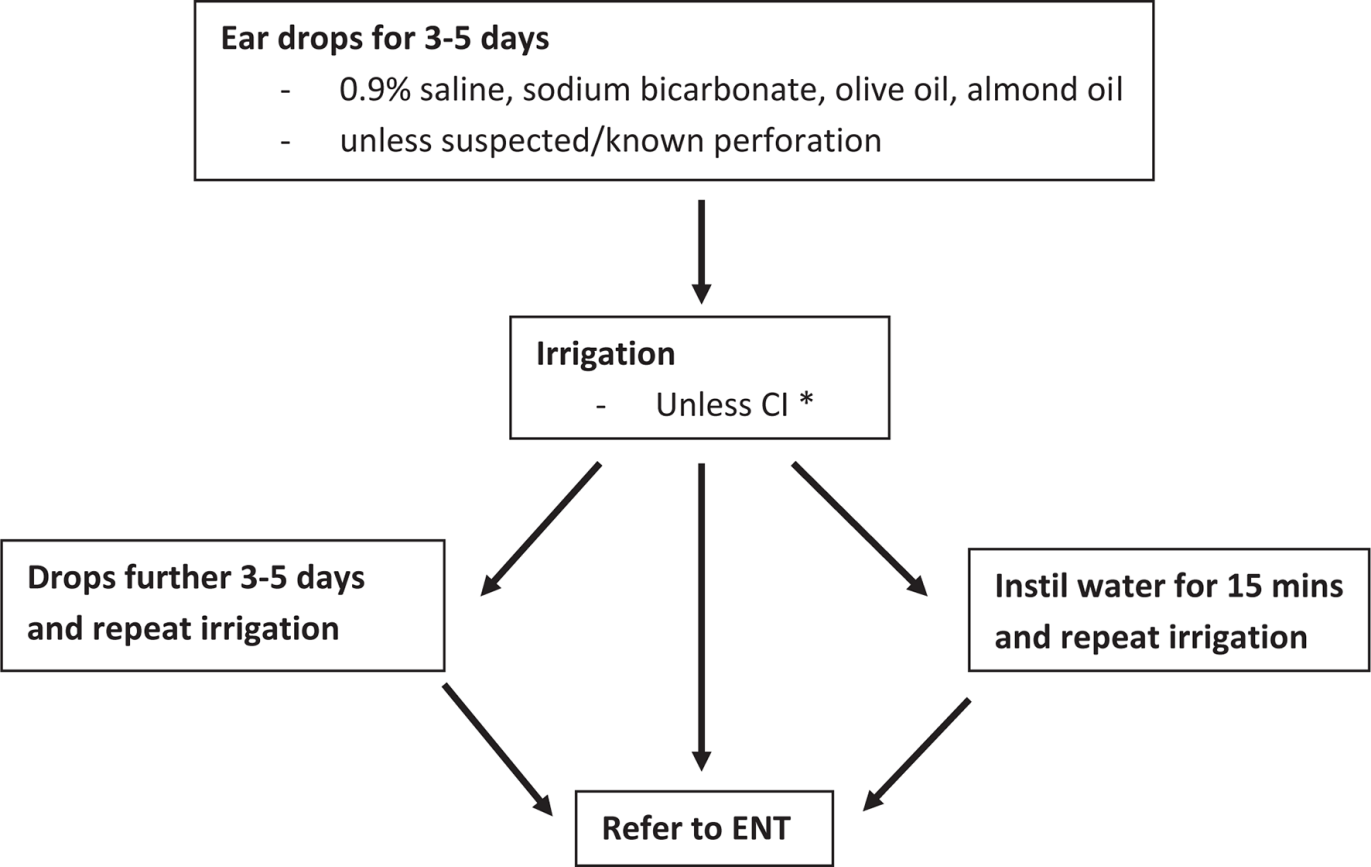
NICE Clinical Knowledge Summaries guidelines on management of earwax.^1,5^
CI = contraindicated. ENT = ear, nose, and throat services. *Previous ear surgery, anatomical abnormality of canal, history of drum perforation/grommet, only hearing ear, infection, under 16 years, previous intolerance of irrigation. Please refer to the full guidelines for comprehensive information on contraindications to irrigation.

Treatment regimes for impacted wax fall into one of four categories: watch and wait, cerumenolytic agents (sodium bicarbonate, olive oil, almond oil, water/saline), irrigation, and manual removal. Often the use of cerumenolytics is combined with either irrigation or manual removal. Current NICE guidance recommends the use of a cerumenolytic for 3–5 days followed by irrigation if symptoms persist (Figure 1).^1^ There is no evidence that using cerumenolytics for longer than this provides additional benefit.^5^

There are no high quality studies comparing irrigation to manual removal of wax. However, it is accepted practice that referral on to specialist ear, nose, and throat (ENT) services for manual removal should be considered when irrigation has failed, or if there are contraindications to irrigation.^1,5^ Contraindications to irrigation include previous ear surgery, anatomical abnormalities of the ear canal (congenital, osteoma, exostosis), history of tympanic membrane perforation (including grommet/ventilation tubes), only hearing ear, under 16 years of age, history of active or recurrent otitis externa, or previous intolerance of irrigation.^1,3^

### Irrigation

Ear irrigation is not without risks. A survey by Sharp *et al* estimated complications occur in 1:1000 ears irrigated, the most common being failure of wax removal (37%), otitis externa (22%), perforated tympanic membrane (19%) and damage to the external auditory canal (15%).^3^ Other cited complications include pain, vertigo, and otitis media, in addition to more serious but thankfully rare sequelae. Wallis and Dovey looked at the rates of primary care treatment injury claims in New Zealand over 5 years; ear syringing and cryotherapy combined caused 13.5% of reported injuries.^6^

Iatrogenic otitis externa following ear irrigation is a complication which has been looked at in more detail. Bruins *et al* estimate that the risk of otitis externa after irrigation is 3%.^7^ Although this seems small, it equates to 69 000 additional cases of otitis externa per year in England and Wales. There is also evidence that malignant otitis externa (caused by *Pseudomonas*) is more common following ear irrigation, particularly in immunocompromised and diabetic patients. Therefore, it has been suggested that irrigation should be avoided in elderly, immunocompromised and diabetic patients.^8^

### Microsuction

Microsuction is the most commonly employed technique for manual removal of wax. The main advantage of this technique is that it is performed under direct vision, and so can be used in clinical scenarios where irrigation would be contraindicated. It is also usually quicker than irrigation, and does not expose the ear canal to moisture. A recent study by Prowse and Mulla looked at the efficacy of microsuction, and found that in a study population of 159 patients, the procedure was successful in clearing the wax in 91% of cases.^9^

There are far fewer reports on the safety of microsuction for removal of earwax. A prospective study of 164 patients in an ENT outpatient clinic found that 55% of patients reported adverse effects, although these were minor and short-lived in most cases.^10^ The most commonly reported symptoms were dizziness, loudness of the procedure, and reduced hearing. An additional finding in this study was that prior use of cerumenolytics significantly reduced the experience of pain and vertigo. Furthermore, a small UK-based study found that the use of audiovisual distraction significantly lowered patients’ perception of pain during microsuction — a potentially useful technique in improving acceptance and patient understanding.^11^

## Discussion

The presence of impacted ear wax has significant implications, not only on hearing, but also with regard to psychological and emotional health, as well as communication and social functioning. Effective treatment of this condition is therefore an important aspect of a holistic approach to managing patients.

Cerumenolytic agents alone are often not fully effective in clearing impacted wax. For many patients, irrigation is a safe and effective approach. However, serious complications can result and the risks increase with immunocompromise, diabetes, and poor compliance, all of which are more prevalent in increasing ageing populations. Contraindications to irrigation are also more prevalent with increasing age, which further reduces the proportion of older patients amenable to irrigation. Microsuction would seem to be well tolerated, and although adverse effects are common they are minor and short-lived. Microsuction also has the ability to provide immediate wax clearance when an urgent clinical need arises, such as sudden hearing loss or where visualisation of the tympanic membrane is necessary for diagnosis. However, there is a paucity of evidence for the clinical and cost-effectiveness of microsuction compared with irrigation, and this is an area highlighted by NICE as a priority for further research.^5^

Given the risks associated with irrigation and microsuction, informed consent for either procedure should always be sought. Providing written information in advance, in the form of patient leaflets, is considered good practice and consideration should be given to using specific consent forms. As a minimum, verbal discussion of risks and consent process should be documented.

Increased provision of microsuction in the community would seem to be a useful approach in improving safety, particularly with an ageing demographic. Referral for microsuction accounts for a significant proportion of the secondary care ENT workload. This presents problems for access, particularly in patient populations who have mobility issues and rely on carers, family, or hospital transport to take them to and from hospital appointments. A recent study in Ireland has shown favourable results with respect to patient satisfaction when comparing a GP-led community microsuction service to traditional hospital-based provision.^12^ The more accessible such treatments can be made, the more equitable the service becomes with regard to disadvantaged patient groups. Community-based services have the potential to do this, as well as of reducing the burden on secondary care services.
